# Desperately Seeking Intersectionality in Digital Health Disparity Research: Narrative Review to Inform a Richer Theorization of Multiple Disadvantage

**DOI:** 10.2196/42358

**Published:** 2022-12-07

**Authors:** Laiba Husain, Trisha Greenhalgh, Gemma Hughes, Teresa Finlay, Joseph Wherton

**Affiliations:** 1 Nuffield Department of Primary Care Health Sciences University of Oxford Oxford United Kingdom

**Keywords:** digital health disparities, video consultations, intersectionality, health inequity, narrative review, digital capital, fundamental cause theory, mobile phone

## Abstract

**Background:**

Digital consultations between patients and clinicians increased markedly during the COVID-19 pandemic, raising questions about equity.

**Objective:**

This study aimed to review the literature on how multiple disadvantage—specifically, older age, lower socioeconomic status, and limited English proficiency—has been conceptualized, theorized, and studied empirically in relation to digital consultations. We focused mainly on video consultations as they have wider disparities than telephone consultations and relevant data on e-consultations are sparse.

**Methods:**

Using keyword and snowball searching, we identified relevant papers published between 2012 and 2022 using Ovid MEDLINE, Web of Science, Google Scholar, and PubMed. The first search was completed in July 2022. Papers meeting the inclusion criteria were analyzed thematically and summarized, and their key findings were tabulated using the Grading of Recommendations Assessment, Development, and Evaluation Confidence in the Evidence from Reviews of Qualitative Research criteria. Explanations for digital disparities were critically examined, and a search was undertaken in October 2022 to identify theoretical lenses on multiple disadvantage.

**Results:**

Of 663 articles from the initial search, 27 (4.1%) met our inclusion criteria. In total, 37% (10/27) were commentaries, and 63% (17/27) were peer-reviewed empirical studies (11/27, 41% quantitative; 5/27, 19% qualitative; 1/27, 4% mixed methods; 1/27, 4% systematic reviews; and 1/27, 4% narrative reviews). Empirical studies were mostly small, rapidly conducted, and briefly reported. Most studies (25/27, 93%) identified marked digital disparities but lacked a strong theoretical lens. Proposed solutions focused on identifying and removing barriers, but the authors generally overlooked the pervasive impact of multiple layers of disadvantage. The data set included no theoretically informed studies that examined how different dimensions of disadvantage combined to affect digital health disparities. In our subsequent search, we identified 3 theoretical approaches that might help account for these digital disparities. Fundamental cause theory by Link and Phelan addresses why the association between socioeconomic status and health is pervasive and persists over time. Digital capital theory by Ragnedda and Ruiu explains how people mobilize resources to participate in digitally mediated activities and services. Intersectionality theory by Crenshaw states that systems of oppression are inherently bound together, creating singular social experiences for people who bear the force of multiple adverse social structures.

**Conclusions:**

A limitation of our initial sample was the sparse and undertheorized nature of the primary literature. The lack of attention to how digital health disparities emerge and play out both within and across categories of disadvantage means that solutions proposed to date may be oversimplistic and insufficient. Theories of multiple disadvantage have bearing on digital health, and there may be others of relevance besides those discussed in this paper. We call for greater interdisciplinary dialogue between theoretical research on multiple disadvantage and empirical studies on digital health disparities.

## Introduction

### Background

COVID-19 has thrown a spotlight on digital health disparities. Before the pandemic, patients with poorer self-reported health, of older age and lower incomes, and from certain minority ethnic groups were less likely to access health care by technological means [1,2]. The proportion of health care consultations conducted remotely (telephone, video, and web-based) increased dramatically during the pandemic [3], chiefly because many face-to-face consultations were canceled owing to the risk of transmitting the virus. Therefore, the shift to remote consultations affected a greater number of patients with increased access burdens because of being disadvantaged through poverty, low health literacy, limited English proficiency (LEP; in countries where English is the main language), or lacking digital skills or devices [4,5].

There is a vast amount of research on health disparities in general; this literature falls into 3 broad categories corresponding to 3 longitudinal phases. The first phase, detection, involves defining health disparities, identifying vulnerable populations, and developing valid measures for studying both. The second phase, understanding why disparities exist, involves identifying factors that explain gaps in health care between vulnerable and less vulnerable groups. The third phase involves the development, implementation, and evaluation of interventions that reduce or eliminate health disparities. These different kinds of research are all relevant to the study of digital disparities as well, although the literature on the latter is currently sparse in both volume and depth.

Digital health is sometimes presented vaguely and futuristically as having the potential to strengthen health systems and public health, improve efficiency, and increase equity in access to health services [6,7]. Video consultations in particular have been extensively researched (often in randomized controlled trials in comparison with face-to-face consultations) and depicted with promissory claims of delivering efficient care without compromising safety or patient satisfaction [8-10]. However, as the pandemic showed, digital *solutions* intended to reduce inequalities may actually widen them [11-13]. Broadly speaking, and with some notable exceptions [14-16], technologies are least frequently and least readily used by limited English–speaking communities, those with low income, and older adults—and especially by those in the *triple jeopardy* of all 3 groups.

Uptake of video consultations, for example, is known to be low among various disadvantaged groups [17]. This and other digital disparities have been explained by multiple factors, including lack of access to technology, low digital literacy, suboptimal internet coverage, and power differentials within the home in terms of who has access to digital devices [18-20].

Several recent publications have proposed strategies to ensure that the emergence of digital services does not exacerbate disparities in access to health care and health outcomes [11,21-23]. In total, 2 broad approaches have been taken. One approach speculates that digitally driven efficiency savings could *free up* staff to attend to the disadvantaged, who would continue to consult in traditional ways. However, there is limited evidence that such savings occur even with telephone consultation services and—to our knowledge—no evidence that they occur with video services (which have higher setup costs and require staff and patients to learn new skills) [24].

Another approach centers on identifying and removing barriers to video access among disadvantaged groups—for example, ensuring that people are equipped, competent, and confident in using the video modality where appropriate. This strategy is founded on an individual deficit model that depicts the disadvantaged as deficient in certain things (eg, knowledge, confidence, and bandwidth) and assumes that these deficiencies can be rectified by specific inputs (eg, training, practice, and digital upgrades). Thus, it tends to overlook the pervasive impact of multiple layers of structural disadvantage. Of particular interest is how key risk factors for digital exclusion such as LEP, poverty, and older age are often mutually reinforcing, an effect that some have called intersectionality [25].

These approaches are discussed throughout this paper, which starts by outlining the aim, scope, and research questions of the review along with definitions of important terms and concepts. We then explain our methodological approach to the review and the details of our methods. Our findings show that, in the relatively sparse literature uncovered on the topic, substantial digital disparities are reported and that this research to date in relation to video consultations has been almost entirely descriptive rather than explanatory. We also describe how we identified 3 candidate theories of multiple disadvantage that could further enhance our understanding of digital disparities. We conclude by proposing 3 candidate theories that may have particular relevance in explaining and helping address digital health disparities in people with multiple disadvantage.

### Aim, Scope, and Research Questions

In this narrative review, we sought to explore how published studies of disparities in digital health consultations have defined, theorized, and empirically tested the concept of multiple disadvantage.

To sharpen our focus in a potentially vast field, we chose to restrict our sample of empirical digital health research to studies of video consultations between patients and health care professionals as this is where the most dramatic differences in digital access have been documented in the literature [8]. In contrast, there has been little research on digital disparities in electronic consultations [26], and research on telephone consultations suggests that digital disparities are less marked [27]. On the basis of findings from our previous work on remote consulting [28], we decided to focus particularly on studies that provided insights relevant to an underresearched group: older adults with low income and LEP.

Our research questions were as follows: (1) How have the intersecting effects of age, socioeconomic status, and LEP been conceptualized, theorized, and studied empirically in relation to digital consultations (especially video)? (2) What interventions have been developed and tested within the context of digital consultations to try to overcome the effects of multiple disadvantage? (3) What were the findings of these studies and how can they help us extend theory and inform future research? and (4) What are the implications for policy and practice?

### Definitions

This review covers a number of closely related terms and concepts, which we define and discuss in Textbox 1.

**Concepts and definitions.**

**Health disparities**

- Defined by the Centers for Disease Control and Prevention of the United States (as this term has almost exclusively been used in the United States) as differences in health status or health outcomes among population groups as a result of—for example—social, economic, racial, or ethnic characteristics [29].

**Health equity**

- Defined by the World Health Organization (WHO) as “the absence of avoidable, unfair, or remediable differences among groups of people, whether those groups are defined socially, economically, demographically or geographically or by other means of stratification” [30]. Others have defined health equity in positive terms as “the attainment of the highest level of health for all people...valuing everyone equally with focused and ongoing societal efforts to address avoidable inequalities, historical and contemporary injustices, and the elimination of health and healthcare disparities” [31]. Thus, reducing health disparities is one aspect of achieving health equity.

**Health inequity**

- Refers to the presence of these avoidable, unfair, or remediable differences [31]. Some authors have distinguished health inequities from *health inequalities*, the latter being disparities that are explained by differences that are not avoidable or remediable (eg, because of age) [32] but, in practice, these terms tend to be used interchangeably.

**Digital health**

- An interdisciplinary field linking technologies (software, hardware, and underpinning infrastructure) and the service models in which they are used [33]. It includes mobile health apps, electronic health records, electronic medical records, wearable devices, remote consultations (by telephone, video, or the web), and remote monitoring of various kinds.
- Digital health includes *telemedicine*, which the WHO set a standardized definition for in 2007 as the delivery of health care services, where distance is a critical factor, by all health care professionals using information and communication technologies for the exchange of valid information for diagnosis, treatment, and prevention of disease and injuries; research and evaluation; and the continuing education of health care providers, all in the interest of advancing the health of individuals and their communities [34].
- The terms *telemedicine* and *telehealth* are often used interchangeably, but telehealth has evolved to encapsulate a broader array of digital health care activities and services, including patient and professional health-related education, public health, and health administration [34].
- *Video consultations* are a specific type of telemedicine involving a video connection.

**Digital divide**

- This constitutes a societal division between those who have the means and capability to make full use of digital technology and those who lack those means for reasons relating to (for example) income, education, or age [35].

**Digital health disparities**

- A concept that emerged recently; refers to inequalities that may be widened when technologies are required for accessing and receiving care. One author has coined the expression “digital inverse care law” to depict how people who are most in need of care (in particular, older people and those experiencing social deprivation) are least likely to access or receive it through digital platforms [36].
- For consistency in this paper, we have chosen to use the term *disparities* rather than *inequities* or *inequalities*.

**Disadvantage**

- Defined as those for whom the social conditions in which they are born, live, and age do not ensure opportunities for them to be healthy and flourish [37]. Disadvantaged people are disproportionately affected by disease, dysfunction, and ill health.
- *Underserved* and *marginalized* populations include people who experience discrimination of any kind and encounter barriers (eg, racial, ethnic, gender, sexual orientation, economic, cultural, or linguistic) to accessing health care goods and services [38]. They tend to receive fewer and lower-quality health care and public health goods and services, have a lack of familiarity with the health care delivery system, face a shortage of readily available providers, and lack access to quality systems of care.

**Intersectionality**

- Refers to the idea that systems of oppression are inherently bound together, thus creating singular social experiences for people who bear the force of multiple systems [25]. It has been defined as “the relationships among dimensions and modalities of social relations and subject formations” [39]. A more specific definition in relation to health disparities is the “intersections of individuals’ multiple identities within social systems of power that compound and exacerbate experiences of ill health” [40], thus recognizing that health is shaped by a multidimensional overlapping of factors such as race, class, income, education, age, ability, sexual orientation, immigration status, ethnicity, indigeneity, and geography.

## Methods

We undertook a narrative review of the literature published from 2012 to 2022 focusing on digital health disparities in disadvantaged groups, with a specific focus on older, low-income, limited English–speaking individuals and on video consultations. Various combinations of search terms, including those regarding age, language, and income, were trialed as part of the initial search strategy but, because of the unique combination of terms being used, they yielded no relevant results. Following discussion with an expert librarian and coauthors, the search strategy shown in Textbox 2 was applied and updated with further terms as the study progressed to reflect the developing field. Although the search range extended back to 2012, only one study in our final sample was published before 2020. Earlier studies were clearly superseded by later work because of technologies having been developed at pace and the evolving field of digital health care. An evaluation of a video consulting service in Scotland by Wherton et al [41] reported that, as late as 2017, the platforms used for video consultations were designed for videoconferencing rather than video consulting and were expensive, clunky, unreliable, and poorly aligned with clinical workflows. A few years later, bespoke video technologies for health care encounters had been developed; they were cheaper, more agile, and better designed around key workflows. Accordingly, studies undertaken before the development of mature, fit-for-purpose technologies were less relevant. Similarly, when looking to the literature for explanations of digital health disparities, little had been published before 2012, and earlier studies reflected challenges that are no longer relevant today, such as website provision and public health dissemination through digital television [42].

We drew in particular on 3 methodological sources. First, we aligned with Greenhalgh et al [43], who highlighted the purpose of narrative review (to achieve clarification and understanding across a broader field of inquiry) and distinguished this from that of quantitative systematic review (to identify, summarize, and synthesize data on a narrowly focused topic, typically through meta-analysis). The latter relies on largely technical processes (eg, data extraction and the use of risk-of-bias tools), whereas the former requires the progressive development and refinement of an argument through interpretative methods.

Second, we engaged with the methodology by Boell and Cecez-Kecmanovic [44] for hermeneutic review, which applies the hermeneutic circle (progressively adding parts to the whole) to secondary research. The hermeneutic review begins with a close reading of key texts—those known to the research team and those identified on an initial scoping search. These texts are mapped and classified according to coherence, adequacy, and relevance (with relevant extracts as quotes) in interim summaries. Some summaries are *deep dives* on specific themes (eg, how a particular theory has been applied). Some are broader but less deep (eg, an early draft of the review findings to be progressively refined as the study unfolds). The researchers move back and forth between further searches and a progressively richer overall summary.

Third, we took note of methodological guidance from a group of journal editors in the health care field [45], who developed a structured critical appraisal tool (SANRA [Scale for the Assessment of Narrative Review Articles]). SANRA defines quality in narrative reviews in terms of strong justification for the importance and aims of the review, a well-described and well-justified literature search, claims backed up by referencing relevant primary studies, the quality of scientific reasoning (including a nonselective approach to inclusion of studies and study designs appropriate to the research question), and appropriate presentation of data.

The following search terms were developed by author 1 and discussed with the coauthors: *remote consultations* (or *virtual consultations* or *video consultations* or *video visits* or *telemedicine* or *telehealth*) and *digital health inequality* (or *digital divide* or *inequity/ies* or *inequality/ies* or *health disparity/ies* or *disadvantage*). These terms were then checked by the expert university librarian. Following some pilot searches, these were developed into formal search strings (Textbox 2).

The search was first completed in July 2022 and then supplemented with an additional search with revised search terms in October 2022. The results of all these searches were combined into a single data set, and irrelevant studies were excluded by screening titles and abstracts. All papers were reviewed by author 1, and each paper was second reviewed by at least one coauthor. Rigor was strengthened by reflexivity and the application of the Grading of Recommendations Assessment, Development, and Evaluation Confidence in the Evidence from Reviews of Qualitative Research checklist [46] to determine confidence in the findings.

To gain familiarity and aid data management, author 1 extracted and tabulated the following information from the included sources: (1) study design, setting, and sample; (2) key findings in relation to health inequalities and web-based care; (3) key issues raised by the authors; (4) authors’ recommendations for how to promote health equity in the context of remote care; and (5) any theories or frameworks used by the authors to study digital disparities.

Following the hermeneutic circle, we worked back and forth between individual papers and our overview of the findings, progressively adding detail. The process of interpretive synthesis was aided by discussions among the coauthors. When we realized that the initial data set of 27 papers included very little theoretical analysis, we conducted a further search for theories of multiple disadvantage that were potentially relevant to digital health disparities and had the potential to add a theoretical depth to our original sample of papers. This second search was deliberately not exhaustive; it included asking experts in the field, using key sources known to the authors, and searching the PubMed database for the term theor* along with the selected terms listed in Textbox 2.

A near-final version of the synthesis was shared with a wider group of peers, including experts in various aspects of health disparity or digital health, and with laypeople with lived experience of accessing care. The synthesis was refined in response to their feedback.

**Search strategy with updated terms.**

**Ovid MEDLINE**

- Search in title, abstract, keywords, and subject headings
- Search limited to the years 2012 to 2022
- Search string: (“remote consultations” or “virtual consultations” or “video consultations” or “video visits” or telehealth or telemedicine) AND (“digital health inequalit*” or “digital divide” or inequit* or inequalit* or “health disparit*” or disadvantag*)

**Web of Science**

- Search in title, abstract, keywords, and subject headings
- Search limited to the years 2012 to 2022
- Search string: (“remote consultations” or “virtual consultations” or “video visits” or telehealth or telemedicine) AND (“digital health inequalit*” or “digital divide” or inequit* or inequalit* or “health disparit*” or disadvantag*)

**Google Scholar**

- General search in Google Scholar
- Search limited to the years 2012 to 2022
- Search string: (“remote consultations” or “virtual consultations” or “video visits” or telehealth or telemedicine) AND (“digital health inequalit*” or “digital divide” or inequit* or inequalit* or “health disparit*” or disadvantag*)

**Sources known to the research team and their networks**

- Key studies already on file
- Asking expert colleagues to recommend sources

**Forward and backward reference searching**

- Identifying highly relevant papers cited by the included papers
- Identifying highly relevant papers that cited the included papers

## Results

### Description of the Data Set

**Figure 1:**
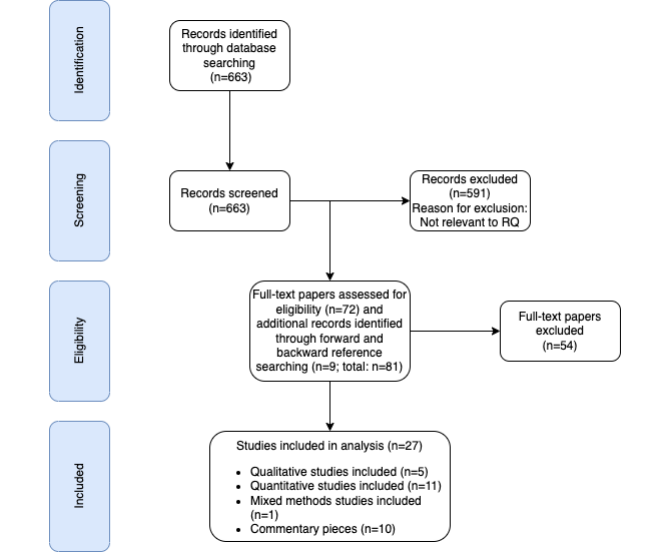
Flow diagram of the search process (adapted from the PRISMA [Preferred Reporting Items for Systematic Reviews and Meta-Analyses] guidelines [47]). RQ: research question.

In this section, we describe the main findings of the empirical studies included in this narrative review (Table 1) as well as key points from commentaries on the theme of inequity of access (Table 2). All the authors of both commentaries and empirical studies offered a list of proposed solutions to digital disparities. These are further summarized and categorized (Textbox 3). Figure 1 presents a PRISMA (Preferred Reporting Items for Systematic Reviews and Meta-Analyses) flowchart demonstrating the number of papers identified, included, and excluded.

The 17 peer-reviewed research papers (summarized in Table 1) comprised 1 (6%) systematic review [48], 1 (6%) narrative synthesis [49], 7 (41%) retrospective audits of medical records [56,57,59-62,70], 3 (18%) quantitative surveys [5,50,58], and 5 (29%) qualitative studies based on semistructured interviews [5,51,52,54,55]. A further 37% (10/27) of the articles (Table 2) commented on others’ research, reflected on findings from clinical practice, or proposed measures to reduce digital health disparities [11,21,22,63-69].

**Table 1:** Characteristics of the empirical studies.

Author, year, and country	Study design	Setting	Sample	Aim of research
Parker et al [48], 2021, United Kingdom	Systematic review	Primary care (any country)	Studies that compared remote and face-to-face consultations (until June 2020)	To explore the impact of remote consultations on use and clinical outcomes in disadvantaged groups
Litchfield et al [49], 2021, United Kingdom	Rapid review and narrative synthesis	UK primary care	Studies that explored various constructs within the 3 domains of the digital divide framework	To identify how this “digital divide” was manifested during the first wave of the pandemic and highlight any areas that might be usefully addressed for practice beyond the pandemic
Chang et al [50], 2020, United States	Quantitative study using rapid response surveys	Small primary care practices in low-income, minority, or migrant areas of New York City	5372 primary care providers contacted in 5 waves	To understand how primary care practices were responding to the COVID-19 pandemic and examine whether telemedicine use and barriers differed based on the socioeconomic characteristics of the communities served
Eberly et al [23], 2020, United States	Quantitative retrospective electronic record audit	Large academic health system	2940 patients who had scheduled a remote consultation	To compare patients who completed telemedicine encounters with patients who were scheduled but did not complete a visit early in the COVID-19 pandemic
Fu et al [5], 2022, United Kingdom	Mixed methods—quantitative survey before and during the pandemic and qualitative data from free-text notes	DOTW^a^ United Kingdom—a third-sector organization serving migrants with drop-in clinics	Migrant patients (survey: n=6268; free-text analysis: n=96)	To understand the living conditions, changes in the service user profile, and needs of vulnerable migrants trying to access health care both before and during the pandemic (when DOTW services moved to telephone)
Stevens et al [51], 2021, United Kingdom	Qualitative interview study (“rapid health needs assessment”)	Across England	42 interviews with people who experienced social vulnerability and faced barriers to accessing health care	To assess the experiences of socially vulnerable people during the COVID-19 pandemic
Kaihlanen et al [52], 2022, Finland	Qualitative semistructured interview study	National study, mostly via third-sector organizations working with vulnerable groups	N=74, including older adults, migrants, mental health service users, high users of health services, and unemployed individuals	To examine the challenges experienced by vulnerable groups using digital health services during the COVID-19 pandemic
Knights et al [53], 2021, United Kingdom	Qualitative semistructured interview study	Urban, suburban, and rural settings in England	48 clinicians, 16 administrative staff, and 17 migrant patients	To understand the pandemic’s impact on recent immigrants and their access to primary health care and implications for vaccine rollout
Alkureishi et al [54], 2021, United States	Qualitative semistructured interview study	Primary care clinics linked to a Chicago academic research center	54 interviews with adult patients and parents of child patients who had web-based visits (March 2020-September 2020)	To understand service users’ perspectives on (1) the definition, causes, and impact of the digital divide; (2) whose responsibility it is to address this divide; and (3) potential solutions to mitigate this
Donaghy et al [55], 2019, United Kingdom	Qualitative semistructured interview study	Scotland primary care	Patients (n=21) and primary care clinicians (n=13)	To explore the views of physicians, nurses, and patients who have experienced a web-based consultation
Rodriguez et al [56], 2021, United States	Quantitative cross-sectional study using electronic health record data	Across primary and specialty care in Boston, Massachusetts	Data from 162,102 patients across 1652 primary and specialty care practices	To use data from a large, integrated health system to determine patient, clinician, clinic, and neighborhood characteristics associated with visit modality
Hsueh et al [57], 2021, United States	Quantitative retrospective cross-sectional study	Primary care across Kaiser Permanente, Northern California	955,352 patient portal self-scheduled primary care telemedicine visits	To test the hypothesis that limited English proficiency would be associated with lower video use compared with telephone, especially among patients without previous video visit experience
Yu and Hagens [58], 2022, Canada	Quantitative cross-sectional web survey	Across Canada	2303 older adult Canadians	To investigate socioeconomic disparities in the demand for and use of web-based visits during the COVID-19 pandemic among older adults in Canada
Schifeling et al [59], 2020, United States	Quantitative retrospective, cross-sectional analysis	2 primary clinics in Colorado	192 appointments reviewed	To determine (1) whether video visits had longer duration, more visit diagnoses, and more discussions than telephone visits in the rapid implementation of telemedicine during the pandemic and (2) whether disparities in visit type existed based on patient characteristics
Sachs et al [60], 2021, United States	Quantitative cross-sectional analysis	Oregon Health and Science University	134,274 ambulatory patients	To evaluate for demographic disparities in the use of telehealth modalities
Broffman et al [61], 2022, United States	Quantitative cross-sectional analysis	Electronic platform across the United States	2847 men	To examine the complex relationship between individual and environmental characteristics, broadband access, device type, and telehealth use as it relates to the digital divide
Zachrison et al [62], 2021, United States	Quantitative retrospective analysis	Electronic health record data across the Northeastern United States	N=1,241,313 individual health records	To describe patient characteristics associated with successful transition from in-person to web-based care and video vs audio-only participation

^a^DOTW: Doctors of the World.

**Table 2:** Characteristics of the commentaries and editorials.

Author, year, and country	Context	Aim of paper
Nouri et al [22], 2020, United States	Commentary emerging from practice, by clinicians at an academic medical center in San Francisco with 3 clinics including an urban “safety-net” service	Discussion of challenges encountered in ensuring equitable access to telemedicine in the early weeks of the pandemic
Ramsetty and Adams [63], 2020, United States	Editorial by directors of free CARES^a^ clinics in South Carolina	To discuss disparities in access to telemedicine among vulnerable patients and evaluate why patients could not access the web-based system at CARES clinics
Mehmi et al [64], 2020, United Kingdom	Commentary on 2 articles, one arguing for the benefits of video consultations and one about health inequalities exposed by the pandemic	Authors criticize a BMJ article for failing to address digital disparities, notably relating to lack of effective internet access in some geographical areas, digital poverty (inability to afford a device or adequate data package), poor digital skills and confidence, refugee and other uncertain citizenship status, and lack of space and privacy at home
Thronson et al [65], 2020, United States	Commentary on an empirical audit showing that the pandemic led to fewer primary care encounters overall and many more conducted remotely	Authors comment that the empirical study failed to capture a key finding from their own clinical experience (supported by audit data): that web-based visits (by video) were rarely taken up by the homeless, limited-English speakers, and those in a “racially diverse safety-net population”
Ramasawmy et al [66], 2021, United Kingdom	Commentary on how the move to digital could increase many well-documented inequities	Summarizes the literature on health inequities, including key reports from the past; warns that these inequities could increase with “digital first” policies; and highlights areas in which existing knowledge and evidence might be translated into cross-sectoral action
Gray et al [67], 2020, United States	Editorial from the Department of Internal Medicine at Ohio State University	To explore the strategies for digital care of vulnerable patients in a COVID-19 world; recommends 5 key strategies to prevent losing touch with vulnerable patients who are alienated by the digital divide
Crawford and Serhal [21], 2020, United States	Commentary summarizing existing literature and offering a new framework	Authors introduce the Digital Health Equity Framework to identify the digital determinants of health and their links to digital health equity; aim is to establish systematic ways to ensure that health inequities are identified and addressed in digital health policies and programs
Rodriguez et al [11], 2020, United States	Opinion piece on digital health equity	To discuss views on how the digital divide should be considered in the implementation of recent policy (21st Century Cures Act)
Eruchalu et al [68], 2021, United States	Commentary emerging from practice (New York City)	To discuss concerns about inequities in digital health access
Gallegos-Rejas et al [69], 2022, Australia	Article proposing a series of practical steps to improve access to telehealth services	Summarizes selected strategies to improve equity of access to telehealth for stakeholder groups: consumers (patients and carers), consumer advocacy groups, health service staff (clinicians), health services (providers), policy makers, funders, and researchers

^a^CARES: Community Aid, Relief, Education, and Support.

**Summary of solutions to digital disparities proposed by authors of primary studies and commentaries.**

**Policy and government**

- Finance and governance
  Extension of temporary waivers by private medical programs for telemedicine beyond the end of the public health emergency declaration
  Develop targeted payment mechanisms to reimburse providers for helping patients adapt to video-enabled telemedicine
  Payment parity between insurers for video and audio visits
  Clarify standards for design of digital health innovations
  Secure funding for projects that address equity and access to health services (including telehealth) with focus on patient experience and acceptance
- Internet access
  Improve distribution of video-enabling devices to those unable to afford them
  Expand device and broadband internet access
  Install free, bookable, soundproofed video booths in community centers, libraries, or physicians’ offices to ensure privacy, with staff available to help with using technology
- Evaluation
  Identify and monitor disparities in access
  Incentivize quality improvement programs based on equity-related outcomes

**Organization and health system**

- Staff training
  Increase system leadership awareness of barriers to telemedicine
  Engage community health workers
  Promote empathy and bedside manner
  Provide clinical telehealth training to all staff
- Service delivery and choice
  Offer digital services to all patients
  Targeted access slots
  On-the-day appointments reserved for marginalized patients
  New models of care such as web-based group consultations
- Technology
  Provide different modality options, including high- and low-technology forms
  Explore technologies that supplement or simulate face-to-face interactions during web-based consultations
  Develop device loan schemes to support those who would benefit from telehealth interventions but do not have access to equipment

**Patients and citizens**

- Translation and communication
  Provide interpreting services
  Translation of relevant documents
  Information and guidance that are more inclusive and relevant to those living in challenging and vulnerable circumstances
  Use of tailored translated texts and text templates to encourage access
- Education and awareness
  Develop programs to improve general and health technology literacy
  Increase public awareness of available resources
- Community engagement and co-design
  Involve marginalized people in co-design and data stewardship
  Develop and evaluate evidence-based health care communication protocols for telemedicine practice to help providers create a patient-centered experience

### Descriptive Findings

Formal research studies on this topic in the early months of the pandemic were few in number, mostly small in size, and conducted rapidly. As such, our data set included no in-depth, theoretically informed empirical studies that had set out to explain how different dimensions of disadvantage combined to affect digital health disparities. During 2020, several clinical authors were moved to write urgent editorials and commentaries on the theme of inequity of access as frontline services shifted from face-to-face to remote modes. The summary of the review findings using the Grading of Recommendations Assessment, Development, and Evaluation Confidence in the Evidence from Reviews of Qualitative Research checklist [46] to determine confidence can be found in Table 3.

**Table 3:** Grading of Recommendations Assessment, Development, and Evaluation Confidence in the Evidence from Reviews of Qualitative Research (GRADE-CERQual) summary of findings.

Summary of review findings	Studies contributing to the review findings	Methodological limitations	Coherence	Adequacy	Relevance	CERQual assessment of confidence	Explanation of CERQual assessment
Low household income, older age, and ethnic minority background (especially limited-English speakers) were all independently associated with lower uptake of video consultations.	Fu et al [5] Moher et al [47] Parker et al [48] Litchfield et al [49] Eberly et al [70] Rodriguez et al [56] Zachrison et al [62] Stevens et al [51] Donaghy et al [55] Ramsetty et al [63] Thronson et al [65] Gray et al [67] Eruchalu et al [68] Mehmi et al [64] Ramasawmy et al [66]	Some concerns about reflexivity [47-49,51,55,63-66, 70], recruitment [5,49,55,65-68,70], and analytical rigor [5,47-49,56,63,64, 67,70]	No concerns—strongly evidenced with qualitative or quantitative data	No concerns [49,51,62,64,67,68] Minor concerns [5,47,48,55,56, 63,65] Moderate concerns [66,70]	No concerns [5,47,49,51,55, 56, 63-68] Minor concerns [48,51,62,70]	High confidence	15 studies contributing data with good coherence and few other concerns
Research into digital health disparities, at least in relation to video consultations, has to date been almost entirely descriptive rather than explanatory.	All studies	Some concerns about reflexivity [47-49,64,65,67, 68,70], recruitment [5,49,56,57, 70], analytical rigor [5,47,48,56,61,63, 66,70], and ethical considerations [56]	No concerns	No concerns [49,51,57,62, 64,65] Minor concerns [5,47,48,55,56, 61,66,68] Moderate concerns [63,67,70]	No concerns	High confidence	All studies contributing data with good coherence and few other concerns
The higher the patient’s social vulnerability index, the more likely the consultation occurred by phone instead of video.	Eberly et al [70] Thronson et al [65] Eruchalu et al [68] Mehmi et al [64] Ramasawmy et al [66]	Some concerns about analytical rigor, recruitment, and reflexivity [64,66,68]	No concerns [64-66,68] Minor concerns—limited sample size and not explored in detail in the study [70]	No concerns [64-66,68] Moderate concerns—limited sample size, not sufficiently “rich” data [70]	No concerns [64-66,68] Minor concerns [70]	High confidence	Only one study contributing data with moderate concerns about sample size
Access to digital health services is hampered by insufficient digital or local language skills.	Parker et al [48] Rodriguez et al [56] Hsueh et al [57] Zachrison et al [62] Donaghy et al [55] Ramsetty et al [63] Thronson et al [65] Gray et al [67] Mehmi et al [64] Ramasawmy et al [66]	Some concerns about reflexivity [48,56,57,64,65,67], analytical rigor [48,56,63,66], and recruitment [57]	No concerns—strongly evidenced with qualitative or quantitative data	No concerns [50-52,54,55, 57-67] Minor concerns [48,56]	No concerns [55,56,63,65-67] Minor concerns [48,61,62,64]	High confidence	Few concerns
Digitization and web-based consultations amplified existing inequalities in access to health care for many migrants because of a lack of digital literacy and access to technology compounded by language barriers and indirect discrimination.	Fu et al [5] Rodriguez et al [56] Stevens et al [51]	Some concerns about recruitment [5,57], analytical rigor [5,56], and reflexivity [56,57]	Minor concerns—strongly evidenced with qualitative data but varying experiences in some studies	No concerns [51] Minor concerns [5,56]	No concerns [5,56] Minor concerns [51]	High confidence	3 studies contributing data with good coherence and few other concerns
Although demand for video consultation services in primary care is likely to rise, for complex or sensitive problems, face-to-face consultations remain preferable.	Broffman et al [61]	Some concerns about analytical rigor	Minor concerns given methodological limitations	Minor concerns	No concerns	Moderate confidence	Only one study contributing data with minor concerns about analytical rigor

### Systematic and Narrative Reviews

Studies published in late 2020 and early 2021 included rapid systematic reviews comparing the impact of face-to-face and remote consultations on a range of predefined variables. Parker et al [48], for example, reviewed quantitative studies from before the pandemic to mid-2020 across socioeconomic and disadvantaged groups in the United Kingdom. A total of 13 studies met their inclusion criteria, and they found that phone consultations were used more by young people of working age, nonimmigrants, and women. Asynchronous web-based consultations were used more by affluent and educated people. Findings in relation to socioeconomic status and ethnicity were inconsistent across primary studies, and none of the included studies reported on quality of care or clinical outcomes.

In a rapid review and narrative synthesis of UK primary care by Litchfield et al [49], 9 studies were identified that explored various constructs within three domains of the digital divide: (1) digital access, within which one study described continuing issues with internet connectivity among vulnerable patients in the United Kingdom; (2) digital literacy, where 7 studies described how ethnic minorities and older adults were less likely to use digital technologies for accessing care; and (3) digital assimilation, where one study described how video technologies can reduce feelings of isolation and another described how older Black men were the most likely group to share information about COVID-19 on social media platforms. This review also found a large number of opinion pieces and editorials on digital disparities.

### Quantitative and Mixed Methods Studies

Nouri et al [22] audited the uptake of remote consultations by demographic group in 3 clinics at an academic medical center in San Francisco. Their paper included a literature review and empirical data from the authors’ own 2 general practices. They showed that the shift to remote consulting had been associated with a decrease in the number of consultations with older adults, those of low socioeconomic status, low–health-literacy groups, limited-English speakers, and Black and Asian minority ethnic groups. The authors offer a framework for addressing inequities, which includes goals such as improving digital literacy and resource barriers, removing health system–created barriers, and advocating changes to support sustained and equitable access.

Rodriguez et al [56] also found lower use of video versus telephone visits among older Black, Hispanic, and Spanish-speaking patients, which extends previous findings that showed decreased telemedicine use among patients with LEP [70]. This study also found that clinicians and practices largely drove this variation in the use of video versus telephone visits, suggesting an important target for intervention.

Hsueh et al [57] similarly found that patients with LEP chose video consultation options less often than those without LEP. However, they also found that, once patients with LEP had video visit use experience, they were not different from patients without LEP in likelihood to reuse video visits.

The survey of small primary care providers in New York City by Chang et al [50] uncovered the percentage of encounters undertaken by telephone, video, or web-based patient portal or face-to-face as well as barriers to remote consultations. A key finding was that the higher a patient’s social vulnerability index, the more likely the consultation occurred by phone instead of video. This was similarly echoed by Broffman et al [61] and Zachrison et al [62], who also found that patients of color, who have historically experienced the greatest access disparities, are significantly more likely to use smartphones to access telehealth compared with a computer. This provides insight into the extent to which low-bandwidth telehealth is accessible under certain conditions.

Although Chang et al [50] conducted a relatively small survey with a low response rate (exact figures not given), their findings align with those of other studies. For example, a retrospective case note review of 80,000 American patients from March 2020 to May 2020 who had completed a telemedicine visit (from a total of 140,000 who had scheduled such a visit) found that, overall, 46% of teleconsultations were by video and 54% were by phone [70]. Factors independently associated with fewer completed telemedicine visits included older age, preference for languages other than English, Asian ethnicity, and Medicaid insurance (indicating low income). Higher rates of telephone consultations were associated with female sex, Black or Latino ethnicity, older age, and lower household income. This was also the case with the study by Schifeling et al [59], where more than half of the older patients did not use video visits, especially if they were from racial or ethnic minority backgrounds or Medicaid beneficiaries, and with the cross-sectional analysis by Sachs et al [60] showcasing seniors, non-English speakers, and Black patients to be more reliant on telephone than video for care. Although these large studies provided important quantitative information about individual risk factors for a failed telemedicine visit (and for a telephone visit over a video visit), they were not designed to explore the interaction between different independent variables. Further theoretical explanations of *how* different risk factors, individually and in combination, contributed to digital inequities in certain vulnerable population groups are necessary to fully understand these data.

Similarly, a mixed methods study based in the United Kingdom by Fu et al [5] found that there was a reduction in video consultations in older users, undocumented migrants, and individuals with poor health, which could mean that those in the greatest need were being excluded, perhaps because of the digital divide evidenced in some groups of migrants before the pandemic. Yu and Hagens [58] echoed similar findings in older adults in Canada, with results highlighting socioeconomic disparities among older adults that could potentially explain this trend, including lower income and education levels that may act as barriers for older adults to acquiring the skills and technologies necessary to use more complex solutions such as video. As Fu et al [5] stated, “those in the greatest need of health care appeared to be less able to access remote services.”

### Qualitative Interview Studies

The qualitative rapid health needs assessment by Steven et al [51] across the United Kingdom found that all groups studied experienced challenges in accessing and following COVID-19 information and government guidance, attributed variously to lack of access to digital technology, lack of translated resources, absent or inadequate tailored support, and inadequate housing. Changes in the organization and delivery of health care services, including closure of outreach and drop-in services, remote consultations, and web-based patient registration, worsened existing barriers to accessing health care.

The semistructured interview study by Kaihlanen et al [52] sought to explore and explain the challenges related to the use of digital health services in Finland through the lens of a digital health equity framework. They found that access to digital health services was hampered by insufficient digital or local language skills.

Knights et al [53] found that digitization and web-based consultations appeared to have amplified existing inequalities in access to health care for many migrants in Finland because of a lack of digital literacy and access to technology compounded by language barriers and indirect discrimination (eg, telephone-only booking services become inaccessible to those without a phone). Health care professionals perceived low digital literacy among migrants and were concerned that web-based consultations resulted in difficulties building trust and risked missing safeguarding cues. These semistructured interviews were conducted by phone, and the sample of migrants was small and skewed; the study was not designed to capture individual, contextualized narratives, and findings were largely impressionistic.

The qualitative study by Alkureishi et al [54] of patient perspectives on the digital divide in US primary care settings explained the concept to participants as follows: “there are people that have and can use technology like computers and the internet. But there are also people that do not have or cannot use this kind of technology. So there is a split or a divide, between people that have and know how to use technology, and those that do not” [61]. These authors found that patients were very aware of the digital divide and described the impacts beyond health care, including employment, education, community and social contexts, and personal economic stability. These participants viewed access to technology and digital skills as important influencers of health disparities.

The clinician-patient relationship was a theme in a qualitative study of patients’ and clinicians’ experiences with video consultations in general practice in Lothian, Scotland [55]. Although the sample size of this study was comparable with that of the study by Knights et al [53], purposive sampling was used in this study to include both sexes, a range of ages and socioeconomic statuses, and those with and without technical problems during their video consultations. The study stated that although the demand for video consultation services in primary care is likely to rise, for complex or sensitive problems that require *touch*, face-to-face consultations remain preferable for patients and clinicians.

### Commentaries

A commentary from 2 directors of free clinics in the United States described how their patients were unable to access their web-based system and offered the solution of a combination of technology and face-to-face services to help address some of these disparities [63]. Their discussion highlighted various upstream societal and social factors (such as mistrust of technology, internet availability regionally, and housing insecurity, to name a few) that were being exposed across hospital systems in the country at a critical time in a public health crisis with no measures in place to address them.

Mehmi et al [64] further discussed how they expected video consultations to increase health inequality if the correct infrastructure is not put in place. Thronson et al [65] considered the underlying cause of this “pandemic of health care inequity” [50] to be access—the disadvantaged simply cannot access telemedicine or home monitoring tools. Ramasawmy et al [66], by contrast, highlighted 3 areas in which existing knowledge and evidence can be translated into cross-sectoral action to avoid further ethnic and digital health inequalities: data and measurement, improved communication, and embedded equality impact.

Gray et al [67] offered 5 strategies to prevent the exacerbation of health disparities for low-income, rural, disabled, ethnic minority, and older adult populations in the United States. They considered sociocultural barriers to digital inclusion, including limited digital skills, low health literacy, disability, low income, and LEP, and structural barriers such as geographic isolation, broadband capacity, and technical hardware.

Crawford and Serhal [21] offered a new digital health equity framework to identify the digital determinants of health and their links to digital health equity, which requires additional evidence and empirical application. Gallegos-Rejas et al [69] proposed practical steps to reduce the digital divide and encourage equitable access to telehealth through improvements in digital health literacy, workforce training in clinical telehealth, co-design of new telehealth-enabled models of care, change management, advocacy for culturally appropriate services, and sustainable funding models.

Another 20% (2/10) of the commentaries, by Rodriguez et al [11] and Eruchalu et al [68], also explored strategies for the digital care of vulnerable patients during the pandemic and further discussed concerns about inequities in digital health access. They reiterated the finding that ethnic minority patients had significantly lower chances of attending telemedicine visits because of inequities in broadband access, lack of available technology, and mistrust of health care professionals.

All the authors of the aforementioned studies offered a list of proposed solutions to digital disparities. These are summarized and categorized in Textbox 3.

Although the changes proposed in Textbox 3 have some face validity, they remain largely untested.

A reviewer of a previous draft of this paper suggested that the taxonomy offered in Textbox 3 (which was our own way of making sense of the data we extracted from papers in our sample) reflected a particular theoretical perspective, namely, the social-ecological framework, which “considers the complex interplay between individual, relationship, community and societal factors” [71]. We agree that this lens could potentially provide an overarching framework within which to synthesize middle-range theories in a future paper.

Our second search identified 3 candidate theories that helped explain the effects of multiple disadvantage identified in our data set: fundamental cause theory, digital capital theory, and intersectionality theory, all of which are discussed in the next section.

The fundamental cause theory and intersectionality theory were the most common theories cited in general health disparity research; digital capital theory was mentioned in studies and commentaries in digital disparity research. Various other theories recurred in the literature but were unhelpful in analyzing our data set. We found the aforementioned theories helpful as (1) intersectionality worked as an overall guiding principle for understanding how people’s lives and characteristics stem from and lead to multiple axes of disadvantage; (2) digital capital theory helped us understand how these axes of disadvantage played out in terms of access to and use of digital resources; and (3) fundamental cause theory sensitized us to the pervasive impact of poverty, which operates through multiple intermediate mechanisms.

## Discussion

### Principal Findings

Findings from our narrative review of digital health disparities in relation to video consultations highlighted that the available literature reports substantial digital disparities. Formal research studies on this topic in the early months of the pandemic were few, mostly small, and rapidly conducted. Research in relation to video consultations to date has been almost entirely descriptive, and our data set included no in-depth, theoretically informed empirical studies that were able to explain how different dimensions of disadvantage combined to affect digital health disparities.

Our narrative review, which focused on video consultations, produced 3 key findings in particular. First, the literature was sparse, comprising only 7% (2/27) of reviews and 63% (17/27) of empirical studies, most of which were published since the start of the COVID-19 pandemic in 2020. Of these studies, most (25/27, 93%) were relatively small and undertaken quickly and under pressure during the pandemic, for example, qualitative studies that comprised one-shot semistructured interviews on convenience samples.

The second finding is that, despite the limitations of the literature, substantial digital disparities were reported [5,22,50-53,56,57,59]. Low household income, older age, and ethnic minority background (especially limited-English speakers) were all independently associated with lower uptake of video consultations [5,50,52,53,58,60,61]. Proposed explanations include lack of digital devices and infrastructure, low health and digital literacy, and inability to understand written resource materials [11,65,66,68]. The disparities found were sometimes dramatic and contrasted strikingly with studies of video consulting undertaken before the pandemic. These studies had framed this as an innovative service model that might increase service efficiency.

The third major finding was that research on digital health disparities, at least in relation to video consultations, has to date been almost entirely descriptive rather than explanatory. All the quantitative studies (audits and surveys) in our data set were designed to generate knowledge of the *association* between particular patient characteristics and the uptake and outcome of video consultations. Although such knowledge is essential in identifying a problem, there is a risk that such studies reduce the complex and interacting aspects of disadvantage to simple variables. Variable-focused research has been criticized by social scientists for oversimplifying context, removing key content (eg, unmeasured variables), overlooking historical path dependencies, and failing to explore how different variables combine and unfold over time to produce complex and sometimes unpredictable outcomes for individuals [72,73]. Explanations generated from such studies and from the superficial and qualitative designs included in our data set tended to couch findings in terms of *barriers* and *enablers*, which were depicted as having more or less fixed effects (negative and positive, respectively) on outcomes. The result is a body of literature that is desperately in need of theorization; for example, none of the 63% (17/27) of empirical studies cited any theory of disadvantage, and none of the well-intentioned ideas listed in Textbox 3 are couched in a well-developed theory of change.

Descriptive, variable-centered research is common when studying health disparities as it allows the so-called social determinants of health (eg, income, education, and gender) to be manipulated by quantitative techniques such as aggregation and correlation. However, such approaches are inherently problematic as they require research participants to be placed into categories that can then be manipulated as variables (*older adults*, *those with low income*, and *ethnic group X*), and findings tend to be presented in terms of what has been called “single-axis” analyses [25].

As Zheng and Walsham [74] have argued, these notions and categorizations do not consider the multifaceted and complex interplay of factors that contribute to digital disparities, nor how a characteristic that disadvantages an individual in one setting may have little adverse impact or even a positive impact in another. In terms of multiple disadvantage, for example, limited-English speakers are more likely to be older and lack basic digital devices and skills [75]. Although these factors may in some cases be mutually reinforcing, *some* older adults from *some* minority ethnic groups may be more likely than White British older adults to live in intergenerational households with good internet connection and family member support—hence, a characteristic (non-White ethnicity) that acts as a *barrier* in one setting may act as an *enabler* in another.

In sum, the current literature on digital health disparities is not only sparse but also in need of richer theorization to generate *explanations* of how different dimensions of disadvantage interact. We have argued elsewhere that the overemphasis in evidence-based medicine on empirical research at the expense of explanatory theory on the *causes* of phenomena (what some have called *EBM+*) may produce impoverished findings [76]. This builds on earlier work emphasizing the crucial importance of theory in selecting *which* hypotheses to test and how when studying disparities [77,78].

We have begun to explore the wider literature to identify theories of multiple disadvantage that have a potential bearing on digital health. In the following sections, we consider the 3 most relevant theories that emerged in our search to date: fundamental cause theory, digital capital theory, and intersectionality theory*.*

### Fundamental Cause Theory

Link and Phelan [79] define a fundamental cause of health disparity as anything that involves resources that influence the extent to which people are able to avoid risks of mortality and morbidity. Socioeconomic status operates as a *fundamental cause* as it (1) involves access to resources (in particular, wealth, income, education, and racial privilege) that allow individuals to avoid diseases and their consequences and (2) affects multiple risk factors (eg, health literacy, quality of medical care, and diet) and disease outcomes that change over time. In short, those with financial resources and high social status can use these resources to avoid disease, seek treatment, and adopt healthy behaviors. The higher risk of heart disease in those of lower socioeconomic status, for example, can be explained by a combination of less access to money, health care, healthy food options, opportunities to exercise safely, and social support. These fundamental *upstream* disparities operate through multiple mediating factors at both the individual level (eg, diet, physical activity, and attendance at screening programs) and the metabolic level (eg, cholesterol, blood glucose, and stress hormones). An intervention that successfully changes one *risk factor* (such as BMI) will have limited impact as the fundamental cause will still operate through other mediating factors. This theory is often invoked when authors talk of the structural determinants of health disparities [80-82]. Although human behavior, lifestyle *choices*, knowledge, and beliefs may *mediate* the link between social determinants and adverse health outcomes, these factors are inadequate as explanatory *causes* of disease.

Fundamental cause theory offers a plausible explanation for the powerful and persistent link between multiple disadvantage and digital disparities. Applied to digital health disparities, it would depict the fundamental cause of these disparities as low socioeconomic status and that this cause operates through *flexible resources* such as access (or lack thereof) to money, knowledge, power, prestige, and beneficial social connections. A key hypothesis based on this theory is that addressing any one proximal cause—for example, by supplying a person with low income with a digital device—will not solve the fundamental problem as, although this *intervening mechanism* may change one *risk factor* (money in this case), it may have limited impact overall as the fundamental cause will continue to cause disparity through other mediating factors such as lack of knowledge (ie, not knowing how to use the digital device given).

### Digital Capital Theory

Bourdieu [83] applied the idea of capital to signify the internal (eg, abilities and attitudes) and external (possessions and attributes) resources that people mobilize to achieve their goals in social life. He highlighted cultural capital as a form of capital that can be accumulated and transformed into other capitals. Digital capital is an extension by Ragnedda and Ruiu [84] of the theory of cultural capital by Bourdieu [83], made up of “both digital competencies and digital technologies.” They argue that digital capital is a form of capital in its own right and is essential for building social, economic, and cultural resources in the digital world that we live in today. Disparities involving digital skills originate in inequalities of access but are mediated by orientations that can only be understood in relation to total life contexts (eg, education, income bracket, age, location, and social support all influence a person’s access to digital technologies and the level of digital skills that they can acquire) [85]. Digital capital is a relatively new concept that scholars have begun to explore empirically using various methodological approaches [86,87]. Digital capital may be estimated, for example, at the individual level by assessing a person’s digital literacy and skills, at the organizational level by measures of digital infrastructure (including the digital competence of personnel), and at the locality level in terms of the quality of the area’s IT infrastructure.

Digital capital theory points us to the hypothesis that traditional forms of capital (such as economic, cultural, and social capital) are converted into digital capital and vice versa and provides the conceptual tools to examine how and to what extent this occurs, thereby illuminating how social inequality relates to digital inequality. If digital spaces—because of social inequality and underlying power structures—become increasingly stratified, there will be significant impacts on how individuals from differing backgrounds gain accumulated forms of capital through the digital realm. In other words, digital capital theory seems to offer an explanation as to why people who already experience health and other disparities find that these disparities widen when services are digitized.

### Intersectionality Theory

The Black feminist scholar Kimberle Crenshaw [25] critiqued traditional studies of Black women’s oppression for offering what she called “single-axis” analyses focusing on either race or gender but failing to integrate the 2 categories. Subsequent authors have extended the original concept of race-gender intersectionality by Crenshaw by adding categories, including nationality, class, age, sexual orientation, and disability [88,89], revealing “crosscutting and mutually reinforcing systems of domination and subordination” that “may construct multiple, uneven and contradictory social patterns” according to Anthias [90]. Intersectionality has been invoked to explain disparities in outcomes within minority ethnic groups in the context of the pandemic [91]. Intersectionality has been studied in many different ways [39]. Most relevant to our own data set is what we call *lived-experience* intersectionality research, which seeks to elucidate (through qualitative methods such as narrative interviews, ethnography, and arts-based methods) the complex and unique experiences of individuals whose identity crosses the boundaries of traditionally constructed groups [40,89].

Intersectionality theory applied to digital health disparities suggests the hypothesis that each individual’s identity and lived experience is unique and multifaceted and that individuals will use (or will not use) digital services based on their own unique identity and circumstances—rather than as members of a single category such as *asylum seeker*, *Black individuals*, or *older adults*. This theoretical approach would support detailed small-scale case studies to see how different aspects of disadvantage interact in individual lives.

### Strengths and Limitations

Although our narrative approach to this review allowed for a comprehensive overview of the wide range of literature spanning digital health disparities, there was no evaluation of selected articles for validity. Nonetheless, quality was not jeopardized as the methodology by Boell and Cecez-Kecmanovic [44] for hermeneutic review, an explicit methodology and accepted standard, was used alongside other methodological guidance for quality judgment. Narrative reviews are also often criticized for the subjective weighing of the studies chosen for the review. We sought to mitigate this through discussion between coauthors and the study team for an investigation focused on using remote care as part of a wider study [28]. To account for any additional selection bias on the authors’ part, all the included studies were second reviewed for relevance.

A major limitation of the review was the sparse and undertheorized nature of the primary literature. We tried to remedy this by beginning to explore the wider literature to identify theories of multiple disadvantage that have a potential bearing on digital health. It should be noted that the 3 theories that we describe in the discussion are not a result of an exhaustive search, and there may be others of relevance. We plan to develop this stream of theoretical research in a future paper.

### Conclusions: Suggested New Research Directions

Studies published since the COVID-19 pandemic began have shown that the move to digital forms of access and care provision has widened health disparities.

This recent literature contrasts strikingly with research on digital health services undertaken before the pandemic, which was largely focused on demonstrating noninferiority of digital modalities in terms of acceptability, safety, and subsequent use of services, often using randomized controlled trials with highly selected samples (stable, compliant, digitally equipped, and digitally confident patients recruited mostly from outpatient settings). The denominator population for digital health research expanded rapidly when such services became the default option for *everyone* for infection control reasons [92], revealing the (previously largely hidden) problem of wide digital disparities linked to multiple aspects of disadvantage. However, as this review has shown, in-pandemic research to date has been descriptive and superficial and has revealed few insights into how digital disparities emerge and play out both within and across categories of disadvantage.

This lack of attention to multiple disadvantage in digital health research to date represents both a unique opportunity and an important challenge for us to engage more curiously and theoretically with this core subject matter. We now need to turn our attention to specifically developing and using interdisciplinary theories of health disparities and technological innovation to inform our studies. Theories, including (but not limited to) fundamental cause theory, digital capital theory, and intersectionality theory, can provide a distinctive foundation for digital health inequality research and serve to guide ongoing research on this topic area. We suggest 3 complementary empirical approaches, as evidenced by previous studies that have conceptualized the aforementioned theories of health disparities.

First, quantitative studies using electronic patient record data should move beyond the current focus on single-axis analyses framed around a reified notion of the *digital divide* to produce category-focused intersectionality research. Such studies would need to be large, prospective, and hypothesis-driven to explore questions about the interaction between different categories of disadvantage; for example, how do gender, education, and ethnicity influence digital disparities in older people? The Digital Health Equity Framework [21] or the eHealth Equity Framework may provide a frame to think comprehensively about multifaceted approaches [93]. Using the eHealth Equity Framework approach in an initial scoping exercise, for example, can illuminate the proximal factors that need to be incorporated to address health inequities while also drawing attention to possible unintended consequences through distal interactions.

Second, qualitative studies of disadvantaged patients’ digital access and experiences should move beyond the one-shot, theory-free semistructured interview on a convenience sample to achieve richly theorized, in-depth longitudinal studies of lived experience in maximum-diversity samples within particular categories of intersectionality. As noted previously, extended narrative interviews and ethnographic techniques should focus on the unique and particular experiences of individuals. Through rich description and the use of literary devices (eg, metaphor and dramatization), such lived-experience studies will reveal how multiple intersections play out over time in members of a particular broad category of intersectionality (eg, people who have low income, are older, and with limited English and multiple health needs) and also, importantly, illustrate the wide diversity of experiences within that group.

Third, based on the findings of this review as well as on the solutions to digital disparities proposed by the authors of primary studies and commentaries, we suggest adding a co-design component to research. Heard et al [40] proposed adapting lived-experience studies to inform the design of inclusive policies and interventions that can take account of the multiple social, cultural, and political contexts within which individual lives are lived and choices are contemplated. They cited the Intersectionality-Based Policy Analysis Framework as a tool to inform the design of such approaches [94]. The framework provides guidance and direction to address the challenges of health inequities across diverse populations in 3 ways. First, it provides an innovative structure for critical policy analysis. Second, it captures the different dimensions of policy contexts, including history, politics, everyday lived experiences, diverse knowledge, and intersecting social locations. Finally, it generates insights, knowledge, policy solutions, and actions that may not be fully captured through equity-focused policy frameworks. This systematic approach will help in designing policy responses that mitigate instead of increase the potential unequal effect of this phenomenon.

Health disparities are already wide and (in many countries) increasing. As society becomes increasingly digitized, the problem is likely to escalate if intersectional disparities are overlooked. This paper, which is intended as the starting point for a wider debate, has outlined a novel and ambitious research agenda. We invite others to help address and extend it.

## Abbreviations

LEP: limited English proficiency
PRISMA: Preferred Reporting Items for Systematic Reviews and Meta-Analyses
SANRA: Scale for the Assessment of Narrative Review Articles
